# TRAF2 as a key candidate gene in clinical hepatitis B-associated liver fibrosis

**DOI:** 10.3389/fmolb.2023.1168250

**Published:** 2023-04-05

**Authors:** Cichun Wu, Jian Zhang, Huiwen Wang, Wei Zhang, Jingqing Liu, Nianqi Zhou, Keyu Chen, Ying Wang, Shifang Peng, Lei Fu

**Affiliations:** ^1^ Department of Infectious Diseases, Xiangya Hospital Central South University, Changsha, China; ^2^ Department of Pathology, Xiangya Hospital Central South University, Changsha, China; ^3^ National Clinical Research Center for Geriatric Disorders, Xiangya Hospital Central South University, Changsha, China

**Keywords:** HBV-associated liver fibrosis, TRAF2, T lymphocyte, human samples, transcriptomics, key candidate gene

## Abstract

**Objectives:** Approximately 240 million individuals are infected with chronic hepatitis B virus (HBV) worldwide. HBV infection can develop into liver fibrosis. The mechanism of HBV-related liver fibrosis has not been fully understood, and there are few effective treatment options. The goal of this study was to use transcriptomics in conjunction with experimental validation to identify new targets to treat HBV-related liver fibrosis.

**Methods:** To identify differentially expressed genes (DEGs), five liver tissues were collected from both healthy individuals and patients with chronic hepatitis B. NovoMagic and Java GSEA were used to screen DEGs and key genes, respectively. Immunocell infiltration analysis of RNA-seq data was, and the results were confirmed by Western blotting (WB), real-time quantitative polymerase chain reaction (RT-qPCR), and immunohistochemistry.

**Results:** We evaluated 1,105 genes with differential expression, and 462 and 643 genes showed down- and upregulation, respectively. The essential genes, such as tumor necrosis factor (TNF) receptor-associated factor-2 (TRAF2), were screened out of DEGs. TRAF2 expression was abnormally high in hepatic fibrosis in patients with hepatitis B compared with healthy controls. The degree of hepatic fibrosis and serum levels of glutamate transaminase (ALT), aspartate aminotransferase (AST), and total bilirubin (TBIL) were positively linked with TRAF2 expression. TRAF2 may be crucial in controlling T lymphocyte-mediated liver fibrosis.

**Conclusion:** Our findings imply that TRAF2 is essential for HBV-induced liver fibrosis progression, and it may potentially be a promising target for the treatment of hepatic fibrosis in hepatitis B.

## 1 Introduction

Approximately 240 million individuals are infected with chronic hepatitis B virus (HBV) worldwide, making it a significant public health issue (Schweitzer et al., 2015; Cornberg et al., 2020). HBV infection can progress and result in liver fibrosis. Excessive extracellular matrix (ECM) deposition is the main pathological feature of liver fibrosis (Kisseleva and Brenner, 2021), which can progress to liver cirrhosis, hepatocellular carcinoma, and liver failure (Harrington et al., 2022; Li et al., 2022; Peiseler et al., 2022). Early detection and treatment of liver fibrosis can prevent further liver damage, reduce morbidity and mortality in patients with liver tumors, and increase the likelihood of successful transplantation (Morrow et al., 2022). Several studies have identified critical molecular targets for the treatment of liver fibrosis and the corresponding targeted medications, such as the nylon X receptor agonist obeticholic acid, which prevents ECM formation (Younossi et al., 2019), and targeted inhibition of lysyl oxidase, which promotes ECM destruction (Chen et al., 2020; Fondevila et al., 2022). However, these drugs are currently being studied in animals or in human clinical trials, and none have been approved for the targeted treatment of liver fibrosis, particularly HBV-related liver fibrosis, to date. Therefore, research into novel mechanisms and therapeutic approaches is urgently needed to find a cure for HBV-related liver fibrosis.

The mechanisms underlying HBV infection that result in liver fibrosis are complex. Hepatic fibrosis in hepatitis B has unique traits in addition to the basic mechanisms of liver fibrosis. Current research suggests that abnormal immune system activation plays a significant role in HBV-associated liver fibrosis (Fisicaro et al., 2020; Baudi et al., 2021). Among them, it is now commonly acknowledged that T lymphocytes’ immune responses, particularly CD8^+^ T cells, play a crucial role in regulating HBV-induced liver fibrosis (Guidotti et al., 1996; Fisicaro et al., 2020; Tan and Schreiber, 2020; Baudi et al., 2021). After a transient, self-limiting HBV infection, the number of T lymphocytes increases significantly, but these T immune cells are usually dysfunctional (Maini et al., 1999; Webster et al., 2004; Hoogeveen et al., 2019). This eventually led to the progression of liver fibrosis. Recent research suggests that dysfunctional CD8^+^ T cells in individuals with chronic HBV infection undergo genetic and epigenetic alterations as a result of continuous antigenic stimulation and a lack of co-stimulatory or cytokine signaling (Bertoletti and Kennedy, 2019). These findings may provide potential new targets for immunotherapy aimed at activating HBV-specific CD8^+^ T cells, which may promise a cure for HBV-associated liver fibrosis (Bertoletti and Kennedy, 2019; Hoogeveen et al., 2019; Du et al., 2022). Furthermore, T-cell depletion can occur during HBV infection (Maini et al., 1999; Webster et al., 2004). Depletion of CD8^+^ T cells during the peak of viremia in experimentally infected chimpanzees delays viral clearance until T cells return, providing the clearest evidence that HBV clearance is mediated in large part by virus-specific CD8^+^ T cells. In addition to triggering a virus-specific humoral response to prevent virus transmission, CD4^+^ T cells help activate and maintain CD8^+^ T cell responses (Yang et al., 2010). However, the molecules that regulate T lymphocytes and thus the progression of HBV-associated liver fibrosis have not been identified.

In this study, Fresh liver tissue from patients with hepatitis B hepatic fibrosis and healthy individuals was used for RNA-sequencing (RNA-seq). Additionally, by using enrichment analysis and experimental confirmation, key molecules in the liver and immune cell infiltration connected to key molecules were identified. Offering a new treatment target for liver fibrosis brought on by HBV is promising.

## 2 Materials and methods

### 2.1 Human liver tissues

Xiangya Hospital in Changsha, Hunan, China, provided 92 liver tissues, including 82 and 10 HBV-induced fibrotic and normal liver tissues, respectively. The fresh liver tissues were obtained intraoperatively or using needle aspiration biopsy. Hepatic hemangiomas or liver transplant recipients donated normal liver tissues and sections between November 2016 and January 2021, while patients with varying degrees of HBV-related liver fibrosis donated fibrotic tissues and sections. Moreover, the Ishak fibrosis score was used for liver fibrosis specimens (Hirschfield et al., 2021; Lawitz et al., 2022). The Ishak1–6 groups had 10, 10, 12, 17, 15, and 18 cases, respectively. Supplementary Table S1 shows the clinical features of the enrolled patients.

The Xiangya Hospital Center, Central South University’s Ethics Review Committee approved the tissue collection [IRB(S) No. 202110223]. All patients provided informed consent.

### 2.2 RNA-sequencing

The datasets used in this investigation are available in the SRA repository (accession number: PRJNA946157). In addition, the RNA-Sequencing (RNA-Seq) data was standardized and screened for DEGs using the R language. The specifics of RNA-seq operation were discussed in our previous study (Wu C. et al., 2021). The clinical characteristics of the patients are shown in Supplementary Table S2.

### 2.3 Gene set enrichment analysis

The Java Gene Set Enrichment Analysis (GSEA) platform was used to carry out the GSEA. Implicated genes were identified as a gene set, and a ranked list and gene set alignment was constructed for each gene ontology (GO) enrichment and Kyoto Encyclopedia of Genes and Genomes (KEGG) pathway. Gene sets were deemed statistically significant if the false discovery rate was <0.25 and *p* < 0.05.

### 2.4 Immunocell infiltration analysis

Using the CIBERSORTx website (https://cibersortx.stanford.edu/), immunocell infiltration analysis of RNA-seq data was carried out.

### 2.5 Western blotting analysis

Liver tissues were lysed using a radioimmunoprecipitation assay buffer containing phosphatase inhibitors and phenylmethylsulfonyl fluoride. The total protein was extracted following centrifugation at 12, 500 *g* for 35 min, and the protein concentration was calculated using a bicinchoninic acid kit. In order to split each protein sample into 30 g for protein blot analysis, 10% sodium dodecyl sulfate-polyacrylamide gel electrophoresis (SDS-PAGE) was used. All proteins were transferred to nitrocellulose membranes to avoid non-specific staining, and for 1 h they were treated with 5% skim milk powder dissolved in 0.1% Tris-Buffed saline/Tween 20. The resultant membranes were gradually incubated with the appropriate primary antibody (TRAF2, 1:1,000, ab126758; Abcam) at 4°C for an entire night before being exposed to the secondary antibody for 2 hours at room temperature. To find the signal, a chemiluminescence imaging apparatus was employed.

### 2.6 Quantitative real-time polymerase chain reaction

TRIzol reagent (Invitrogen, Grand Island, NY, United States) was used to extract total RNA for quantitative real-time polymerase chain reaction (RT-qPCR) following the manufacturer’s instructions. Reverse transcriptase (1 g) was used to create cDNA from RNA samples (Promega, Madison, WI, United States). Target gene expression levels were also determined using an RT-qPCR method using 2 Taq PCR Master Mix (Qiagen, Beijing, China) and primers (Supplementary Table S3) using an ABI PRISM 7500 Sequence Detection System (Applied Biosystems, Foster City, CA, United States). The negative control was distilled water instead of cDNA. The relative mRNA expression of the target genes was evaluated using the comparative Ct (threshold cycle) technique, as it was previously reported (Livak and Schmittgen, 2001).

### 2.7 Histological examinations

Following liver histology testing, a pathologist from Central South University’s Xiangya Hospital used Masson trichrome staining to assess the pathological abnormalities in the liver tissues. The Ishak fibrosis score was used to determine the degree of liver fibrosis.

### 2.8 Biochemical assays

An automated chemical analyzer was used to measure the levels of regular blood, liver function, and coagulation in serum (Olympus, Tokyo, Japan). Biochemical tests were conducted in the clinical laboratory of Xiangya Hospital of Central South University.

### 2.9 Immunohistochemical and immunofluorescence staining

Anti-TRAF2 antibody was used to detect TRAF2 by immunohistochemistry in paraffin-embedded human liver samples (1:100, ab126758; Abcam). For the negative controls, liver tissues from healthy individuals and those with liver fibrosis were stained with anti-mouse IgG subtype control (ab37415, Abcam). Liver portions were pre-blocked for 10 min after dewaxing. Subsequently, the sections were sealed with 2% goat serum, coated with 1% hydrogen peroxide to neutralize endogenous peroxidase activity, and heated with sodium citrate in an autoclave to repair the antigen. The primary antibody was then left to sit in the slides overnight at 4°C. Secondary goat anti-rabbit immunoglobulin antibodies and immunoperoxidase were applied to the slides (ABC kit, Vector, United States). Anti-TRAF2 antibody (1:50, ab126758; Abcam), anti-CD45 (1:200, ab40763; Abcam); anti-CD3 (1:50, sc-20047; Santa Cruz); anti-CD4 (1:50, sc-19641; Santa Cruz); and anti-CD8 (1:50, sc-1177; Santa Cruz), DAPI were used to label the cell nuclei to identify the cellular localization of TRAF2 in human liver fibrosis liver tissue. Following the manufacturer’s recommendations, the slides were cleaned twice with PBS and inspected using a confocal microscope (ZEISS, Oberkochen, Germany). At 10 HPF/section (around 400), the quantity of positive cells was finally counted.

We used the positive cell ratio during the data analysis, which is the number of positive cells divided by the total amount of cells (Benonisson et al., 2019).

### 2.10 Statistical analysis

Data are displayed as mean ± SE. Prism was used for the analysis. Additionally, a *t*-test was used to examine student data for statistical differences, as indicated by the legend. Statistics were considered significant at *p* < 0.05.

## 3 Results

### 3.1 Data normalization

**FIGURE 1:**
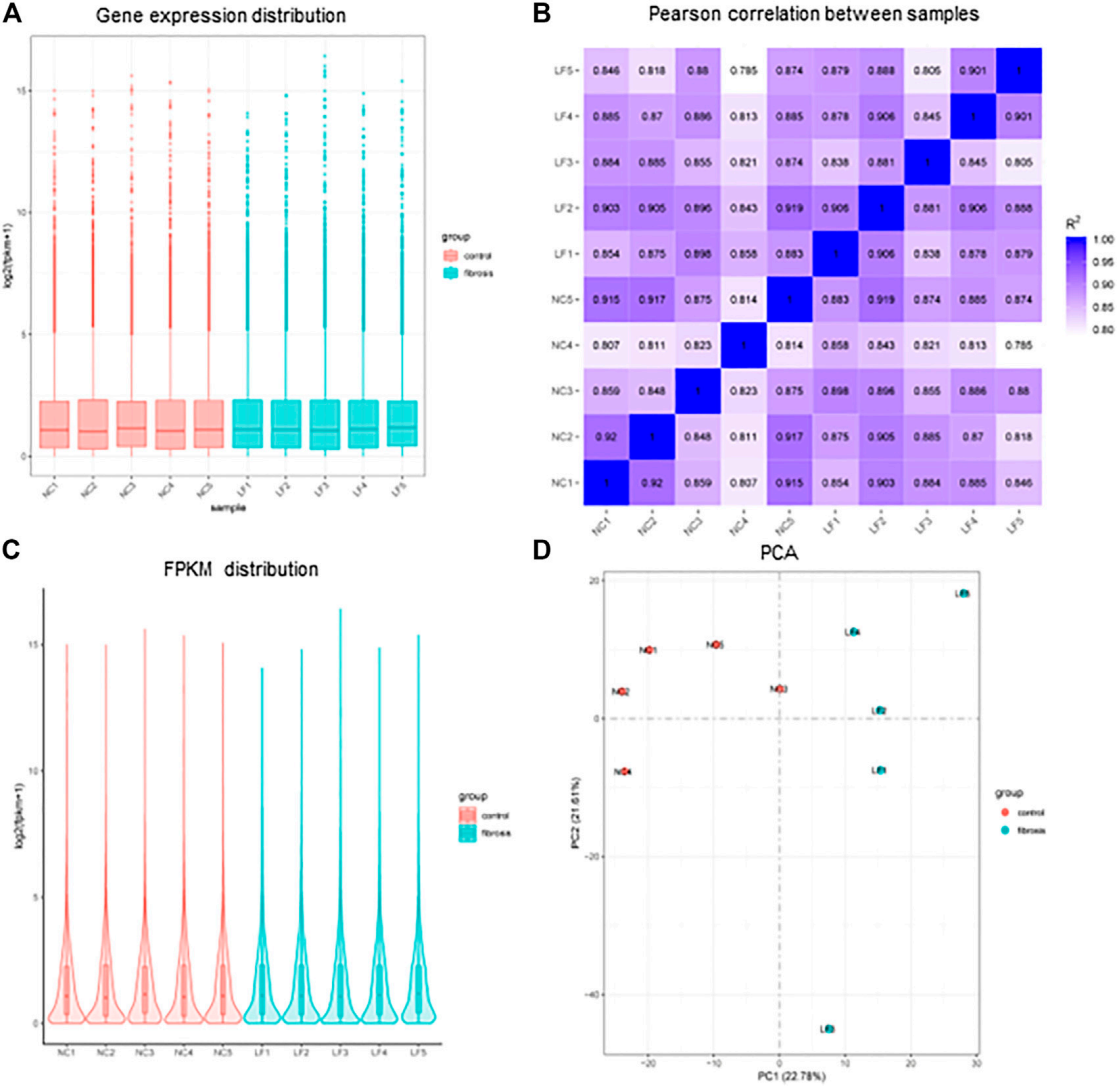
Distribution of sample expression situation after normalization. **(A)** Gene expression distribution of each sample. **(B)** Plot of Pearson correlation coefficient between samples. **(C)** Fragments Per Kilobase of exon model per Million mapped fragments (FPKM) distribution of each sample. **(D)** Principal Component Analysis (PCA) diagram. n = 5, LF, liver fibrosis; NC, normal control.

The primary goal of standardization was to remove technical and systematic variabilities from the data to compare the different samples. The overall distribution of gene expression and values after normalization of the microarray data were consistent between the liver fibrosis (n = 5) and control groups (n = 5) (Figures 1A, B). The use of Pearson’s correlation was good between the samples, with R2 values > 0.8 (Figure 1C). In addition, the biological variability among the various samples was assessed using the principal component analysis plots (Figure 1D).

### 3.2 DEGs identification

**FIGURE 2:**
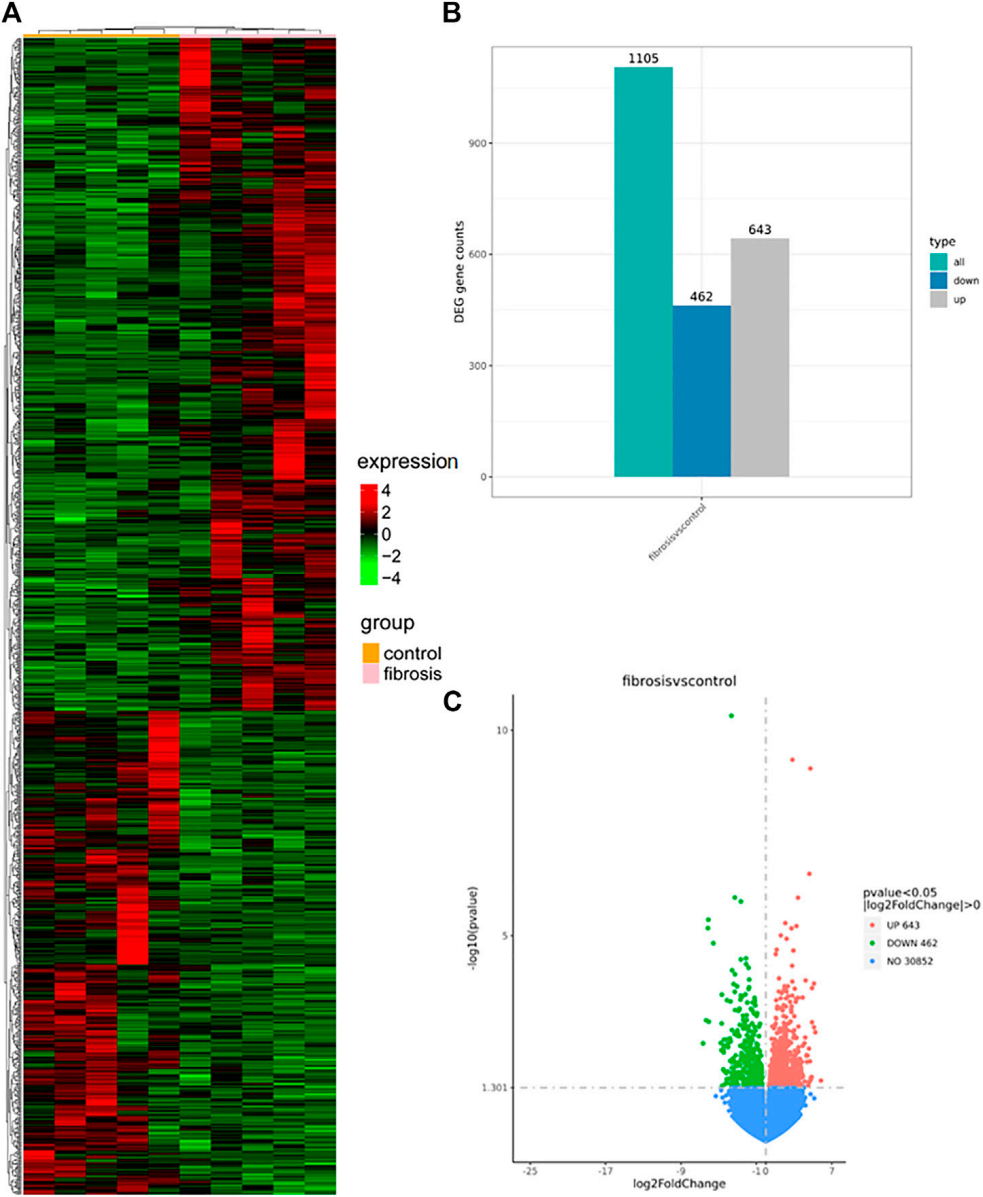
Clustering plot **(A)**, histogram **(B)**, and a volcano plot **(C)** were generated using the NovoMagic platform. **(A)** Clustering map illustrating up- and down-regulated mRNA expression in the HBV-associated liver fibrosis group, with red and green representing selected up- and down-regulated genes, respectively. **(B)** Histogram mapping of mRNA expression in the HBV-associated hepatic fibrosis group. Gray and blue represent selected up- and down-regulated genes, respectively. **(C)** The volcano plot shows the up- and down-regulation of mRNA expression in the HBV-associated hepatic fibrosis group, with red and green representing selected up- and down-regulated genes, respectively. DEGs, differentially expressed genes.

*p* < 0.05, │Log Fold change│>0 based on the NovoMagic platform, screened for DEG. We analyzed 1,105 DEGs, of which 643 and 462 were up- and down-regulated, respectively. Figures 2A–C show a clustering plot of DEGs, histogram, and volcano plot, respectively.

### 3.3 Functional enrichment analysis of DEGs to screen core genes

**FIGURE 3:**
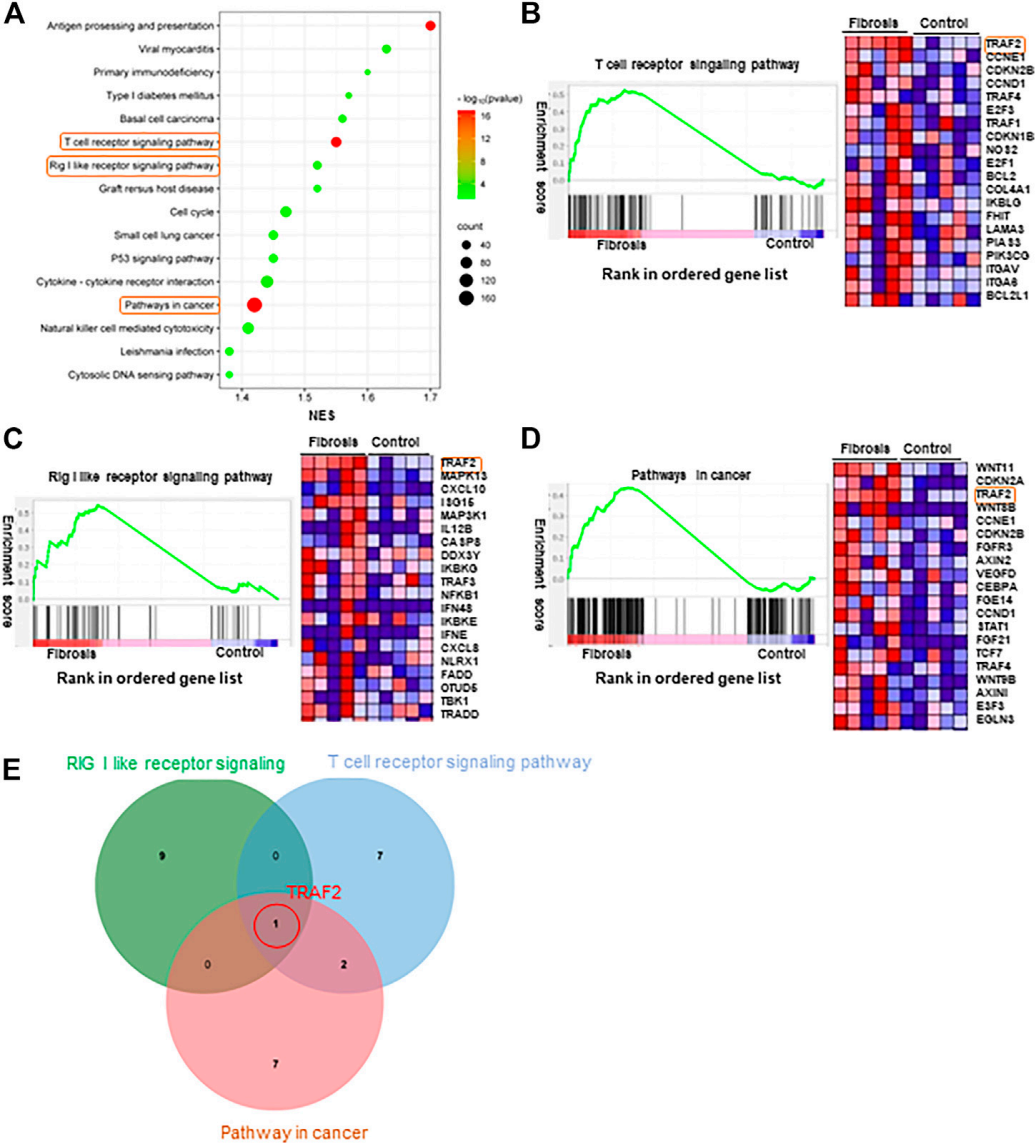
GSEA enrichment analysis. **(A)** Significantly enriched genomes were set to default cutoff values of *p*-value <0.05 and FDR <0.25. KEGG pathway in GSEA. **(B)** T cell receptor signaling pathway KEGG pathway in GSEA. **(C)** The Rig-I-like receptor KEGG pathway in GSEA. **(D)** Pathways in cancer KEGG pathway in GSEA. **(E)** Venn diagram showing the top 10 genes expressed in the three pathways of **(B–D)** cross.

**FIGURE 4:**
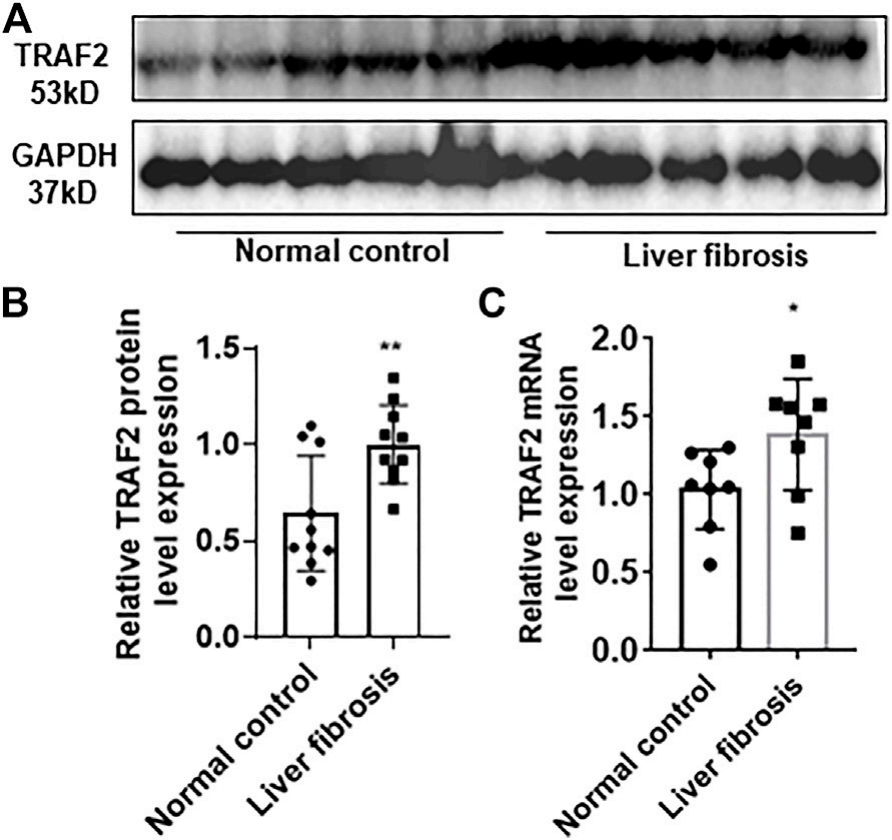
Characterization of TRAF2 expression in hepatitis B liver fibrosis. **(A)** Schematic representation of TRAF2 protein expression in the hepatitis B liver fibrosis and control groups by using Western blotting, n = 10. **(B)** Statistics of **(A)** plots by Image J. **(C)** TRAF2 mRNA expression in the hepatic fibrosis and control groups of hepatitis B by using RT-qPCR, n = 10. *p < 0.05, **p < 0.01. TRAF2, TNF receptor-associated factor 2.

To further investigate the differences in biological processes between the two groups, GSEA analysis was performed, and significantly enriched gene sets were set to default cutoff values of *p* < 0.05 and false discovery rate (FDR) < 0.25. Additionally, the KEGG pathway in GSEA was enriched to include multiple pathways (Figure 3A). Previous research has shown that the three pathways “Rig I like receptor”, “Pathways in cancer”, and “T cell receptor signaling pathway” (Figures 3B–D) play an important role in hepatitis B fibrosis and subsequent development into hepatocellular carcinoma (Yao et al., 2021; Chen et al., 2023; Zhang et al., 2023). We investigated the mRNA expression levels of the top5 genes in these three pathways and discovered that TRAF2 and C-X-C motif chemokine ligand 10(CXCL10) expression was significantly increased, while Wnt family member 8B(WNT8B) and TRAF4 expression was significantly decreased (Figure 4C, Supplementary Figure S1). Furthermore, we took the first ten intersections of these three pathways and discovered the core gene TRAF2 (Figure 3E).

### 3.4 Individuals with hepatitis B liver fibrosis exhibit markedly increased TRAF2 expression in their liver tissues

In order to further validate enhanced TRAF2 expression in hepatic fibrosis, 10 normal liver tissues and 10 liver tissues with hepatic fibrosis in hepatitis B were acquired. TRAF2 protein expression was significantly higher in the hepatic fibrosis group than in the control group, according to Western blotting (WB) (Figures 4A, B).

### 3.5 TRAF2 and the severity of liver fibrosis caused by HBV are positively associated

**FIGURE 5:**
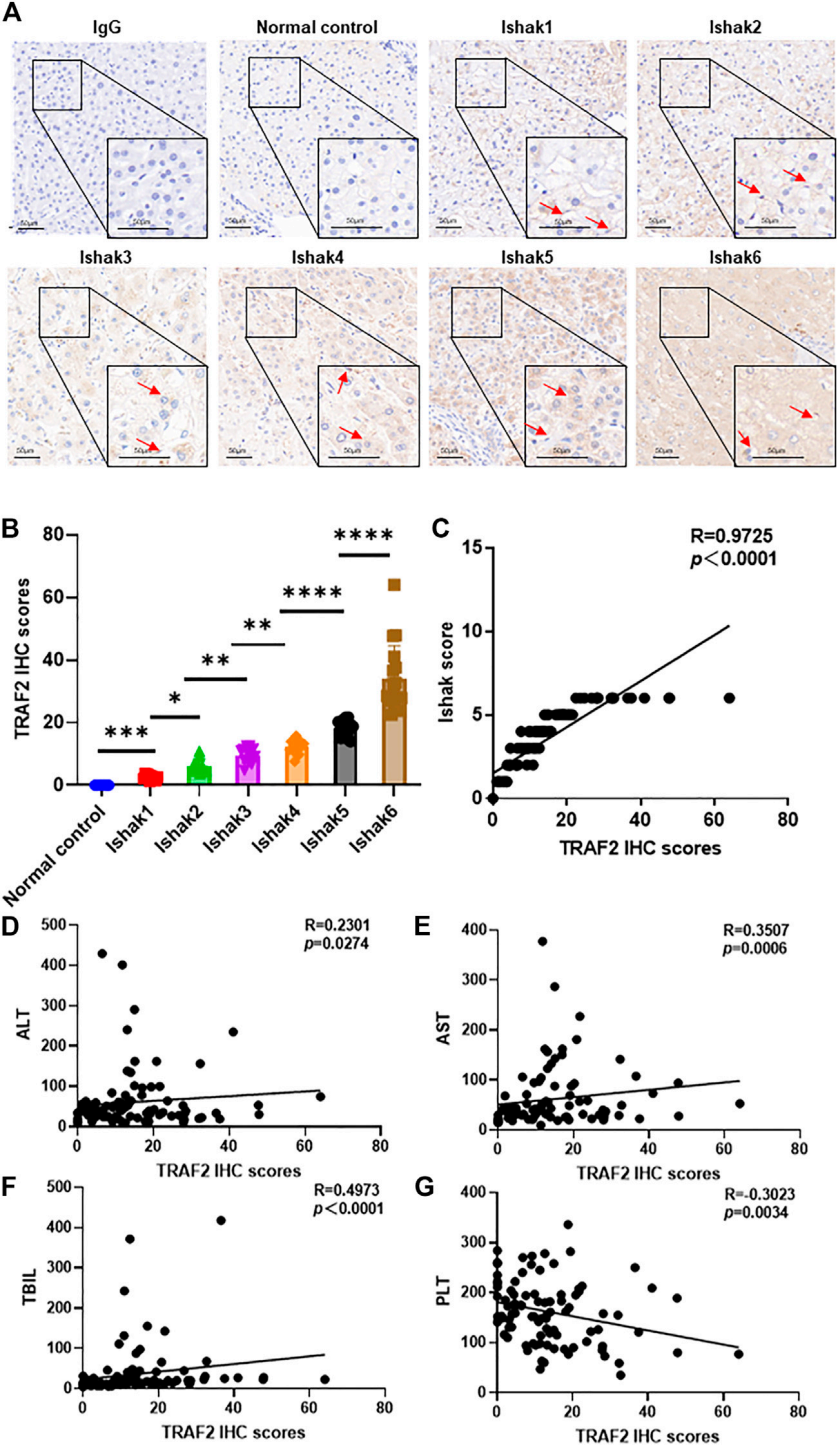
Correlation of TRAF2 with HBV-induced liver fibrosis severity. **(A)** Based on the Ishak score (Ishak1–Ishak6), immunohistochemistry was used to assess the expression of the TRAF2 protein in hepatic fibrosis caused by HBV. IgG was used as the negative control. Scale bar: 50 µm. **(B)** TRAF2 expression was analyzed using Image J software. **(C)** Spearman’s correlation analysis of the relationship between TRAF2 expression and the severity of HBV-induced hepatic fibrosis. **(D–G)** Spearman’s correlation study of the relationship between ALT **(D)**, AST **(E)**, TBIL **(F)**, and PLT **(G)** and TRAF2 expression. Data are expressed as mean ± SEM or median M (P25, P75). Two independent samples t-tests or the Mann-Whitney U rank-sum test are used to evaluate the differences between groups. *p < 0.05; **p < 0.01; ***p < 0.0001; ****p < 0.00001. TRAF2, TNF receptor-associated factor 2; ALT, alanine aminotransferase; AST, aspartate aminotransferase; TBIL, total bilirubin; PLT, platelet.

Furthermore, we collected diseased liver samples from hepatitis B liver fibrosis patients, and immunohistochemical analysis revealed that TRAF2 was considerably increased in these tissues compared with the control group (Figure 5A). Based on the immunohistochemical data of TRAF2, TRAF2 expression was substantially correlated with the severity of liver fibrosis and increased with it (*p* = 0.0001, R = 0.9745, Figures 5B,C). Interestingly, TRAF2 was inversely connected with platelet (PLT) (*p* = 0.0034, R = 0.3023) count and positively correlated with alanine aminotransferase (ALT) (*p* = 0.0274, R = 0.2301), aspartate aminotransferase (AST) (*p* = 0.0006, R = 0.3507), and total bilirubin (TBIL) (*p* < 0.0001, R = 0.4973) (Figures 5D–G). Additionally, other clinical serum markers were not significantly correlated (Supplementary Figure S2).

### 3.6 TRAF2 May promote the development of hepatitis B liver fibrosis through immune infiltration of T lymphocytes

**FIGURE 6:**
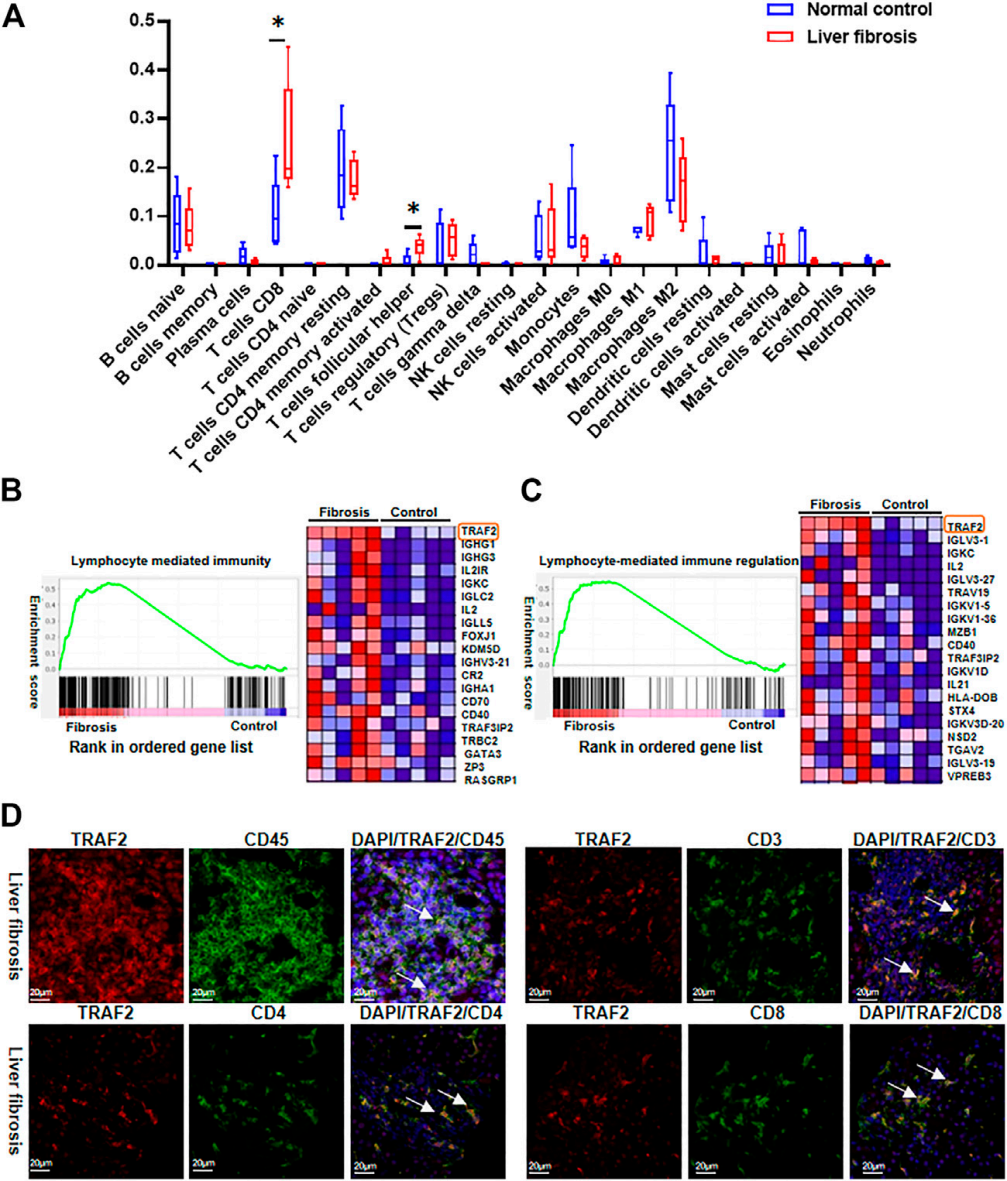
Characterization of TRAF2 expression in immune cells. **(A)** RNA-seq data was analyzed for immunocell infiltration using the CIBERSORTx website. **(B)** Lymphocyte-mediated immunity GO enrichment in GSEA. **(C)** Regulation of lymphocyte-mediated immune regulation GO enrichment in GSEA. **(D)** According to the Materials and Methods, an immunofluorescence (IF) study was performed to determine the location of TRAF2 in liver tissues on a cellular level. The representative double immunofluorescence pictures show that HBV-fibrotic liver tissues express CD45 (green), CD3 (green), CD4 (green), CD8 (green), and TRAF2 (red). Scale bar: 20 μm *p < 0.05. TRAF2, TNF receptor associated factor 2; CD45, cluster of differentiation 45; CD3, cluster of differentiation 3; CD4, cluster of differentiation 4; CD8, cluster of differentiation 8.

It is well known that immune cell infiltration plays an important role in HBV-associated liver fibrosis (Meng et al., 2022; Zander et al., 2022; Zhong et al., 2022). Using CIBERSORTx, we compared immune cell infiltration between the two groups and discovered that many immune cells were altered, with CD8^+^ T cells and T cell follicular helpers most significantly altered (Figure 6A), This result indicates that T lymphocytes play a vital role in the development of hepatic fibrosis in hepatitis B.

We further employed GSEA to examine the data to see whether TRAF2 is related to the immunological infiltration of T lymphocytes, and default cutoff values of *p*-value < 0.05 and FDR<0.25 were used to identify highly enriched gene sets. In the GO enrichment of GSEA, it was discovered that TRAF2 expression was up-regulated in “lymphocyte-mediated immunity” and “lymphocyte-mediated immune regulation” and was situated in the first position among DEGs (Figures 6B,C).

Additionally, immunofluorescence co-localization was used to identify TRAF2 expression in different cells in liver tissues with HBV-associated liver fibrosis. The cell markers CD45, CD3, CD4, and CD8 were used to identify leukocytes, total T cells, CD3^+^ T cells, CD4^+^ T cells, and CD8^+^ T cells, respectively. TRAF2 positive staining was overlapped with CD45, CD3, CD4, and CD8 (Figure 6D). This suggests that TRAF2 is important for T-lymphocyte-dominated immune regulation in hepatic fibrosis in hepatitis B and may influence hepatic fibrosis development through it.

TRAF2 activates the TAK1/P38 or TAK1/NF-kB signaling pathways to activate the “Rig-I-like receptor signaling pathway,” which is responsible for the release of the pro-inflammatory factors IL-8 and TNFα (Supplementary Figure S3A). According to a subsequent RT-qPCR study, the mRNA levels of TNFα and IL8 were significantly higher in the liver fibrosis group compared to the control group (Supplementary Figure S3B). This shows that TRAF2 also may accelerate the development of HBV-related liver fibrosis by releasing the inflammatory factors TNFα and IL8 through the TAK1/P38 or TAK1/NF-kB signaling pathways.

## 4 Discussion

The pathogenesis of HBV-associated liver fibrosis is complex, and liver fibrosis is a major risk factor for developing hepatocellular carcinoma (Newberry et al., 2021). Furthermore, chronic portal hypertension caused by liver fibrosis is a major cause of clinical complications such as ascites, hemorrhage, and hepatic encephalopathy (Rockey et al., 2009; Häussinger et al., 2022). However, treatments for HBV-related liver fibrosis are currently limited (Tan and Schreiber, 2020; Kim et al., 2022). Exploring the pathogenesis of HBV-induced liver fibrosis is therefore critical for identifying key therapeutic targets. Our study screened 1,105 DEGs at the genomic level for further bioinformatic analysis, and we identified TRAF2 as a potential target for hepatic fibrosis treatment in hepatitis B.

Tumor necrosis factor (TNF) receptor-associated factor-2 (TRAF2) is a TRAF protein family member (Shi and Sun, 2018) expressed in cytotoxic T cells, macrophages, natural killer (NK) cells, and neutrophils (Yang and Sun, 2015). TRAF2 was initially discovered as a protein that binds to and interacts with various TNF receptor superfamily receptors in addition to the TNF receptor 2 (Kukimoto-Niino et al., 2022; Siegmund et al., 2022). TRAF2 plays a significant role in inflammatory signaling and malignancies by serving as an E3 ubiquitin ligase and scaffolding protein that controls nuclear factor kappa B (NF-κB) signaling and apoptosis activation (Shi and Sun, 2018; Li et al., 2020; Wu W. et al., 2021; Xu et al., 2021; Qian et al., 2022). NF-κB is a crucial transcriptional regulator of the inflammatory response, and mediators released from Kupffer cells after hepatocyte damage (such as IL-1β) activate signaling pathways, such as NF-κB in hepatic stellate cells (HSC). NF-κB activation causes HSC to activate and secrete chemokines that make HSC more sensitive to TGF, which promotes liver fibrosis (Luedde and Schwabe, 2011; de Souza Basso et al., 2021; Ebrahim et al., 2022; Wang et al., 2022). Further evidence supports that TRAF2 is involved in collagen I transport *via* tyrosine kinase-interacting kinase (TNIK), and thus in the progression of liver fibrosis in mice (Buchl et al., 2022). However, TRAF2 has not been studied in liver fibrosis caused by HBV. Therefore, utilizing liver samples from individuals with hepatitis B-induced liver fibrosis, we examined the protein and mRNA levels of TRAF2. TRAF2 expression in hepatic fibrosis groups was significantly higher than in the control group. More intriguingly, TRAF2 showed a significant correlation with the severity of hepatic fibrosis in hepatitis B, suggesting that TRAF2 may also play an important role in human hepatic fibrosis in hepatitis B.

In our study, the number of immune cells increased in the hepatitis B liver fibrosis group, with CD8^+^ T cells increasing the most. Subsequently, immunofluorescence co-localization was used to identify TRAF2 in CD3^+^, CD4^+^, and CD8^+^ T cells, suggesting that TRAF2 may contribute to hepatitis B liver fibrosis *via* immune T cells. TRAF2 regulates T cell immunity by maintaining the activity of the Tpl2-ERK survival signaling axis in effector and memory CD8^+^ T cells (Xie et al., 2021). Moreover, TRAF2 inhibits IL-6-mediated signal transduction and transcription factor 3 (STAT3) activation, which is required for developing IL-17-secreting CD4^+^ TH17 cells (Nagashima et al., 2018). Moreover, TRAF2 and TRAF5 bind to glycoprotein 130 kDa (gp130) constitutively and inhibit IL-6-driven signaling in CD4^+^ T cells (Kimura et al., 2018). Our study showed that the lymphocyte-mediated immunomodulatory pathway TRAF2 was significantly elevated and ranked first in the GSEA enrichment analysis of genome sequencing. These findings imply that TRAF2 may play a role in the development of HBV-related liver fibrosis by T lymphocytes. However, the specific mechanism remains to be further elucidated.

We discovered that TRAF2 activates the “Rig-I-like receptor signaling pathway,” which is responsible for releasing the pro-inflammatory cytokines IL-8 and TNFα by activating the TAK1/P38 or TAK1/NF-kB signaling pathways. Furthermore, the liver fibrosis group had significantly higher TNFα and IL8 mRNA levels than the control group. However, more studies are needed to confirm that TRAF2 may accelerate the progression of HBV-associated liver fibrosis by encouraging the release of the inflammatory factors TNFα and IL8 through the TAK1/P38 or TAK1/NF-kB signaling pathways.

To the best of our knowledge, this study showed for the first time that patients with hepatic fibrosis in hepatitis B had considerably higher hepatic TRAF2 expression levels. TRAF2 was favorably linked with circulating indicators of liver injury and the degree of hepatic fibrosis in patients with hepatitis B. Our findings also showed that immunological T cells were the major cell type expressing TRAF2. These results indicate that TRAF2 is a key player in the initiation and development of hepatitis B liver fibrosis and is promising as a possible target for therapeutic intervention.

## Data Availability

The datasets presented in this study can be found in online repositories. The names of the repository/repositories and accession number(s) can be found below: https://www.ncbi.nlm.nih.gov/, PRJNA946157.

## Abbreviations

HBV, hepatitis B virus; DEGs, differentially expressed genes; TRAF2, tumor necrosis factor (TNF) receptor-associated factor-2 (TRAF2); GSEA, gene set enrichment analysis; CccDNA, covalently closed circular DNA; ALT, alanine aminotransferase; AST, aspartate aminotransferase; TBIL, total bilirubin; PLT, platelet; TNFα, tumor necrosis factor *α*.
