# Supplementary material for: TRAF2 as a key candidate gene in clinical hepatitis B-associated liver fibrosis
Source: Front Mol Biosci. 2023 Apr 5;10:1168250. doi: 10.3389/fmolb.2023.1168250 (PMC10113534; doi:10.3389/fmolb.2023.1168250)
Supplement: Supplementary file 1 [file Table1.DOCX]

Supplementary Material

TRAF2 As a Key Candidate Gene in Clinical Hepatitis B-associated Liver Fibrosis

**Cichun Wu^1^, Jian Zhang^1^, Huiwen Wang^1^, Wei Zang^1^, Jingqing Liu^1^, Nianqi Zhou^1^, Keyu Chen^1^, Ying Wang^2^, Shifang Peng^1,3*^, Lei Fu^1,3*^**

^1^Department of Infectious Diseases, Xiangya Hospital Central South University, Changsha 410008, China

^2^Department of Pathology, Xiangya Hospital Central South University, Changsha 410008, China

^3^National Clinical Research Center for Geriatric Disorders, Xiangya Hospital Central South University, Changsha 410008, China

*** Correspondence:**Corresponding Author: Prof. Lei Fu, Department of Infectious Diseases, Xiangya Hospital, Central South University, Changsha 410008, Hunan, China. Email: [fulei92@126.com](mailto:fulei92@126.com). Prof. Shifang Peng, Department of Infectious Diseases, Xiangya Hospital Central South University, Changsha 410008, Hunan, China. Email: [sfp1988@csu.edu.cn](mailto:sfp1988@csu.edu.cn).

# Supplementary Figures and Tables

## Supplementary Tables

**Supplementary Table 1.** Clinical characteristics and biochemical results of healthy controls and patients with HBV-associated hepatic fibrosis were collected during the experimental validation.

|  | Control(n = 10) | Ishak1(n = 10) | Ishak2 (n = 10) | Ishak3 (n = 12) | Ishak4 (n = 17) | Ishak5 (n = 15) | Ishak6 (n = 18) |
| --- | --- | --- | --- | --- | --- | --- | --- |
| Male | 6(60%) | 5(50%) | 6(60%) | 6(50%) | 9(53%) | 7(47%) | 12(67%) |
| Female | 4(40%) | 5(50%) | 4(40%) | 6(50%) | 8(47%) | 8(53%) | 6(33%) |
| Age(years) | 37.6 ±6.64 | 45.8 ±7.33 | 47.3 ±8.84 | 50.5 ±10.02 | 47.76 ±6.70 | 44.40 ±8.29 | 52.33 ±6.08 |
| ALB (g/L) | 42.87 ±5.09 | 39.12 ±4.95 | 40.99 ±5.76 | 40.53 ±4.026 | 38.09 ±6.47 | 38.09 ±6.15 | 41.59 ±7.14 |
| ALT(u/L) | 11.25(9.4,23.25) | 43.2(34.1,54.78) | 37.75(32.48,51.43) | 40.85(24.53,57.52) | 52.4(33,136.8) | 64.2(31.5,100) | 33.6(22.68,56.35) |
| AST(u/L) | 17.8(16.05,22.55) | 40.15(29.25,44.65) | 38.55(31.47,58.05) | 35.50(24.15,51.30) | 55.60(38.55,139.8) | 87.80(48.80,150.5) | 34.45(27.62,62.45) |
| TBIL (µmol/L) | 10.05(5.63,14.83) | 10.65(8.88,20.17) | 11.90(8.10,28.32) | 16.05(7.55,20.95) | 21.60(11.25,42.35) | 17.50(13.30,87.90) | 22.55(19.20,28.32) |
| TBA(µmol/l) | 5.20(2.95,8.05) | 7.65(2.83,22.08) | 5.15(2.83,16.88) | 8.60(4.78,10.20) | 16.80(5.90,129.20) | 15.20(4.10,75.70) | 5.40(2.45,16.20) |
| PT(s) | 12.03 ±1.66 | 13.53 ±1.46 | 12.53 ±1.86 | 12.34 ±1.55 | 14.76 ±3.76 | 13.03 ±0.97 | 12.98 ±2.37 |
| PTA (%) | 100.68 ±13.58 | 90.01 ±11.69 | 95.609 ±14.808 | 98.649 ±12.37 | 84.966 ±23.07 | 98.07 ±10.90 | 90.44 ±15.86 |
| INR | 0.97 ±0.12 | 1.08 ±0.12 | 1.03 ±0.15 | 0.99 ±0.13 | 1.19 ±0.3 | 1.05 ±0.83 | 1.07 ±0.17 |
| WBC(x10 × 9/L) | 5.24 ±1.45 | 5.67 ±2.0 | 6.69 ±1.89 | 5.88 ±1.174 | 7.36 ±4.42 | 5.0 ±2.01 | 5.38 ±2.32 |
| PLT(x10 × 9/L) | 217.3 ±45.98 | 149.7 ±30.21 | 186.5 ±35.78 | 189.83 ±63.57 | 118.76 ±48.52 | 179.6 ±75.18 | 128.06 ±59.46 |

Note: The Shapiro–Wilk test for normality was used to analyze measurement data, and data with a normal distribution were expressed as mean ±SEM. Two independent samples t-test was used to evaluate the differences between groups. Data with a non-normal distribution were presented as the median M (P25, P75), and the Mann–Whitney U rank-sum test was used to assess the differences between groups. Count data were presented as the number of cases (percentage), and the χ2 test was used to analyze the differences between groups. Hepatic fibrosis severity was divided into Ishak1–Ishak6 six stages based on the Ishak score. HBV, hepatitis B virus.

**Supplementary Table 2.** Clinical characteristics and biochemical results of healthy controls and patients with HBV-associated liver fibrosis were recorded during RNA-Seq.

|  | NC1 | NC2 | NC3 | NC4 | NC5 | LF1 | LF2 | LF3 | LF4 | LF5 |
| --- | --- | --- | --- | --- | --- | --- | --- | --- | --- | --- |
| Gender | male | male | male | Female | female | male | male | male | female | female |
| Age(years) | 36 | 42 | 48 | 32 | 46 | 35 | 37 | 45 | 43 | 39 |
| ALB (g/L) | 47.9 | 48.6 | 48.4 | 53.3 | 52.6 | 34.9 | 42 | 35.5 | 46.2 | 44.2 |
| ALT(u/L) | 19.2 | 10.6 | 12.2 | 13.2 | 11.2 | 44 | 19.9 | 11.2 | 63.1 | 55.9 |
| AST(u/L) | 27.4 | 18.7 | 16.8 | 22.1 | 26 | 41.2 | 22.5 | 16.2 | 45.1 | 39.1 |
| TBIL (μmol/L) | 4.4 | 5.4 | 9 | 10.9 | 7.8 | 17.8 | 30.9 | 14.9 | 7.4 | 21.9 |
| PT (s) | 11.6 | 13 | 10.9 | 11.6 | 12.9 | 13.4 | 16.3 | 12.9 | 13.3 | 14.3 |
| PTA (%) | 120.7 | 105.4 | 137.6 | 119 | 99 | 97 | 70.7 | 106.5 | 102.2 | 91.3 |
| INR | 0.9 | 1.02 | 0.94 | 1.01 | 1.03 | 1.28 | 1.01 | 1.01 | 1.04 | 1.12 |
| WBC(x10*9/L) | 6.7 | 4.9 | 4.5 | 5.4 | 6.8 | 3.6 | 5.1 | 5.2 | 5.7 | 4.6 |
| PLT(x10*9/L) | 255 | 242 | 211 | 185 | 192 | 146 | 143 | 141 | 185 | 153 |

Note: NC, normal control; LF, liver fibrosis.

**Supplementary Table 3. qPCR sequences**

| Name | **Sequence** |
| --- | --- |
| Human-GAPDH(Forward Primer) | ATGACCCCTTCATTGACC |
| Human-GAPDH(Reverse Primer) | GAAGATGGTGATGGGATTTC |
| Human-TRAF2(Forward Primer) | TGGAAGCCAAGTACCTGTG |
| Human-TRAF2(Reverse Primer) | CCTTCTTCATATATGCCCTCGT |
| Human-AMPK13(Forward Primer) | TGAGCCGACCCTTTCAGTC |
| Human-AMPK13(Reverse Primer) | AGCCCAATGACGTTCTCATGC |
| Human-CXCL10(Forward Primer) | GTGGCATTCAAGGAGTACCTC |
| Human-CXCL10(Reverse Primer) | TGATGGCCTTCGATTCTGGATT |
| Human-ISG15(Forward Primer) | CGCAGATCACCCAGAAGATCG |
| Human-ISG15(Reverse Primer) | TTCGTCGCATTTGTCCACCA |
| Human-MAP3K1(Forward Primer) | CATCAGGTCGCACAGTGAAAT |
| Human-MAP3K1(Reverse Primer) | TCAGGGCTATATGGTGAGAAGC |
| Human-WNT11(Forward Primer) | GGAGTCGGCCTTCGTGTATG |
| Human-WNT11(Reverse Primer) | GCCCGTAGCTGAGGTTGTC |
| Human-CDKN2A(Forward Primer) | GATCCAGGTGGGTAGAAGGTC |
| Human-CDKN2A(Reverse Primer) | CCCCTGCAAACTTCGTCCT |
| Human-WNT8B(Forward Primer) | CCGACACCTTTCGCTCCATC |
| Human-WNT8B(Reverse Primer) | CAGCCCTAGCGTTTTGTTCTC |
| Human-CCNE1(Forward Primer) | GCCAGCCTTGGGACAATAATG |
| Human-CCNE1(Reverse Primer) | CTTGCACGTTGAGTTTGGGT |
| Human-CDKN2B(Forward Primer) | GGGACTAGTGGAGAAGGTGC |
| Human-CDKN2B(Reverse Primer) | CCATCATCATGACCTGGATCG |
| Human-CCND1(Forward Primer) | GCTGCGAAGTGGAAACCATC |
| Human-CCND1(Reverse Primer) | CCTCCTTCTGCACACATTTGAA |
| Human-TRAF4(Forward Primer) | TATTGGGCCTGCCTATCCG |
| Human-TRAF4(Reverse Primer) | CAAAACTCGCACTTGAGGCG |
| Human-TRAF1(Forward Primer) | TCCTGTGGAAGATCACCAATGT |
| Human-TRAF1(Reverse Primer) | GCAGGCACAACTTGTAGCC |
| Human-TNFα(Forward Primer) | CCTCTCTCTAATCAGCCCTCTG |
| Human- TNFα(Reverse Primer) | GAGGACCTGGGAGTAGATGAG |
| Human-IL8(Forward Primer) | TTTTGCCAAGGAGTGCTAAAGA |
| Human-IL8(Reverse Primer) | AACCCTCTGCACCCAGTTTTC |

## Supplementary Figures

**Supplementary Figure 1.** T cell receptor signaling pathway, Rig-I-like receptor signaling pathway, and the pathways in cancer top 5 genes except TRAF2 mRNA expression in the hepatic fibrosis and control groups of hepatitis B by using RT-qPCR, n = 8. *P < 0.05, **P < 0.01. MAPK13, mitogen-activated protein kinase 13; CXCL10, C-X-C motif chemokine ligand 10; ISG15, ISG15 ubiquitin like modifier; MAP3K1, mitogen-activated protein kinase 1; WNT11, wingless-type MMTV integration site family, member 11; CDKN2A, cyclin dependent kinase inhibitor 2A; WNT8B,Wnt family member 8B;CCNE1, cyclin E1; CDKN2B, cyclin dependent kinase inhibitor 2B; CCND1, cyclin D1; TRAF4, TNF receptor associated factor 4; TRAF1, TNF receptor associated factor 1.


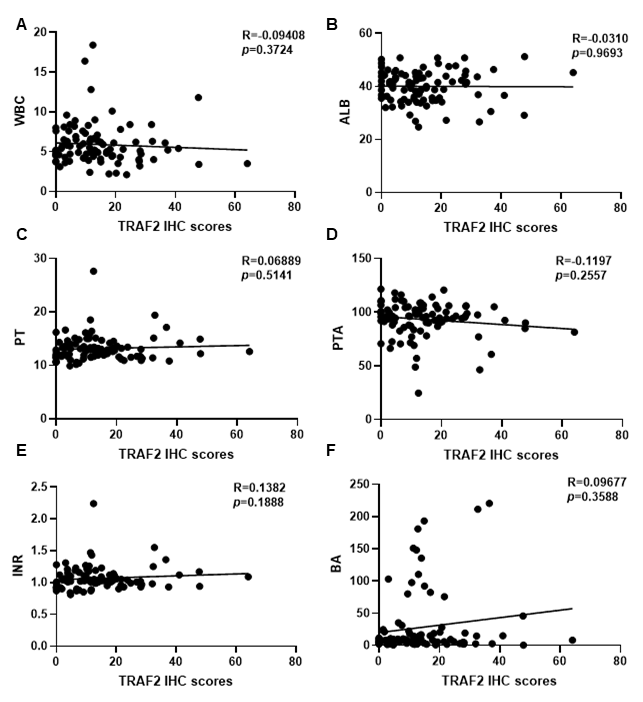


**Supplementary Figure 2.** Spearman’s correlation analysis of the relationship between clinical characteristics and TRAF2 expression in HBV-associated liver fibrosis. Spearman’s correlation analysis of the correlation between TRAF2 expression and (A) WBC, (B) ALB, (C) PT, (D) PTA, (E) INR, and (F) BA. TRAF2, TNF receptor-associated factor 2; ALB, albumin; WBC, white blood cell; PT, prothrombin time; PTA, plasma thromboplastin antecedent; INR, international normalized ratio; BA, bile acids.


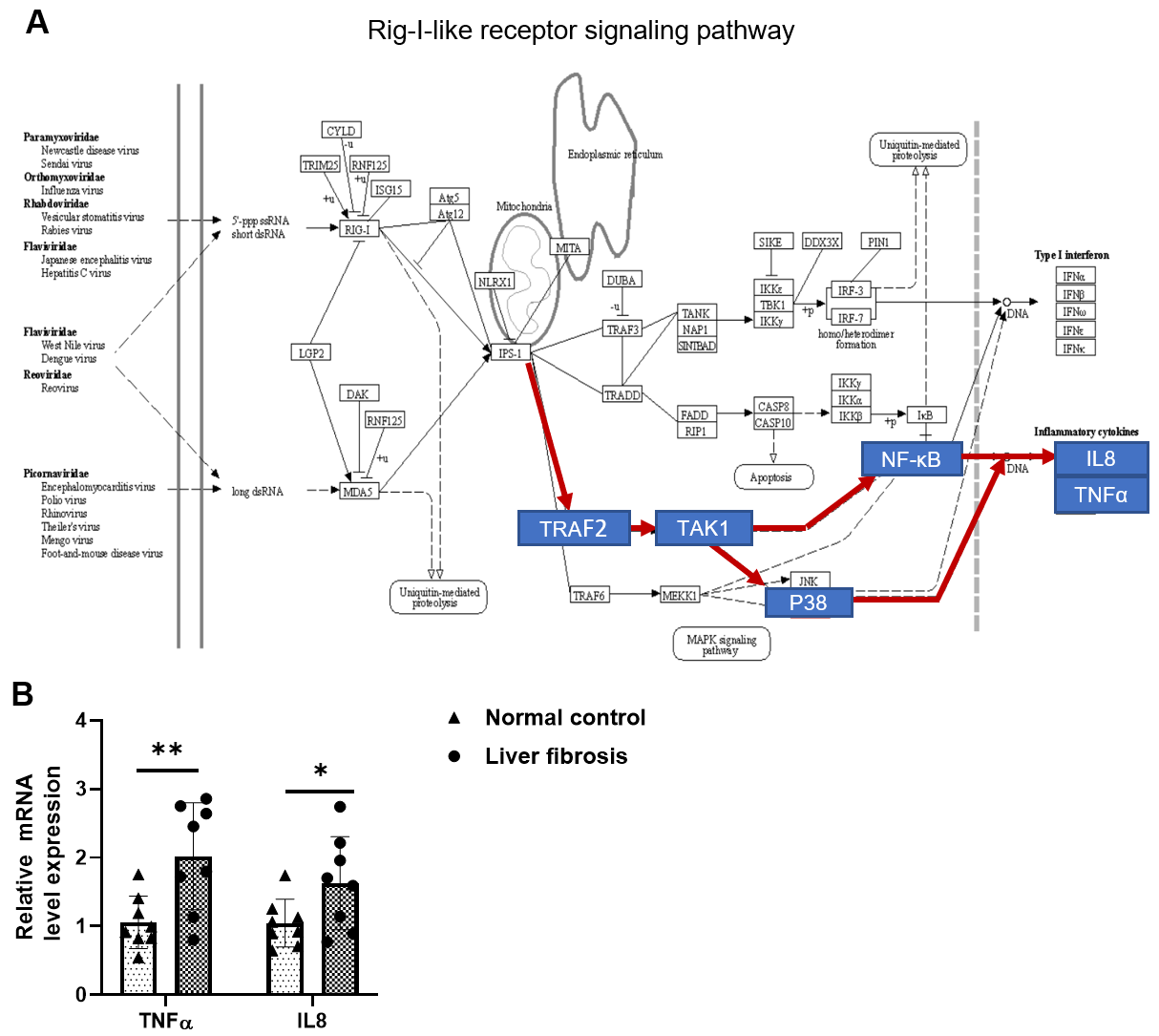


**Supplementary Figure 3.** A, “Rig-I-like receptor signaling pathway” KEGG pathway diagram. B, TNFα and IL8 mRNA expression in the hepatic fibrosis and control groups of hepatitis B by using RT-qPCR, n = 10. *P < 0.05, **P < 0.01. TRAF2, TNF receptor-associated factor 2; IL8, interleukin 8; TNFα, tumor necrosis factor α.
